# The Production of Malignant Primary Hepatic Tumours in the Rat by Feeding Dimethylnitrosamine

**DOI:** 10.1038/bjc.1956.15

**Published:** 1956-03

**Authors:** P. N. Magee, J. M. Barnes

## Abstract

**Images:**


### THE PRODUCTION OF MALIGNANT PRIMARY HEPATIC TUMOURS IN THE RAT BY FEEDING DIMETHYLNITROSAMINE

P. N. MAGEE AND J. M. BARNES

*From the Medical Research Council, Unit for Research in Toxicology, Woodmansterne Road, Carshalton, Surrey.*

Received for publication January 6, 1956

IN a previous paper (Barnes and Magee, 1954) some toxic properties of dimethylnitrosamine (DMN) were described. It was found that rats and other animals suffer severe liver damage after the administration of DMN in doses of the order of 25 mg. per kilogram body weight, either orally or parenterally. The liver lesion was an extensive centrilobular type of necrosis, involving all lobules, followed by evidence of regeneration in surviving animals. An outstanding feature of the necrosis was its very haemorrhagic character and the liver lesion was frequently accompanied by massive haemorrhagic ascites and bleeding into the gastro intestinal tract.

These features of the acute liver necrosis were similar to those produced by the senecio alkaloids in rats (Davidson, 1935). A recent paper (Schoental, Head and Peacock, 1954) showed that senecio alkaloids also produced liver tumours if they were administered over longer periods. It seemed possible, therefore, that the chemically much simpler molecule DMN might also produce liver tumours in rats. The present paper describes the results of a long-term feeding experiment in which the compound was added to the diet of rats. A smaller and shorter experiment on rabbits was also performed. In the rats a very high incidence of hepatic tumours occurred, many with metastatic spread, but in the rabbits no tumours were observed. In no instance were primary tumours found in organs other than the liver.

### MATERIAL AND METHODS

Male and female albino rats and cross-bred male rabbits were used. The basal diets were M.R.C. Diet 41 (Bruce and Parkes, 1949) for the rats and M.R.C. Diet 18 (Bruce, 1947) for the rabbits. The diet was given in powder form. DMN was made up as a solution in arachis oil so that 10 ml. added to 1 kg. of powder gave the required concentration.

The food intake of the rats was measured for each cage of 5 animals by filling the container each day and measuring the unconsumed residue.

The animals were weighed weekly and observed each day when their food was being measured. Post mortems were carried out on all animals that were either found dead or killed by coal gas when seriously ill. Tissues were fixed in Helly's fluid and formol-saline, embedded in paraffin and sectioned at $5\mu$. All sections were stained with haematoxylin and eosin as a routine and selected material was stained by Mallory's connective tissue method, Gomori's silver impregnation, and

Perl's prussian blue reaction for iron. Frozen sections of formol fixed material were stained for fat with Sudan IV.

### RESULTS

*First experiment*

The results of this experiment have been briefly reported previously (Barnes and Magee, 1954) and are included here in greater detail for comparative purposes.

Groups of 6 rats were fed 200, 100 and 50 parts per million (ppm.) DMN respectively. Those receiving 200 ppm. died in less than 5–6 weeks, and at autopsy on 3 rats the livers were small and pale, but regular in outline. There was no intraperitoneal haemorrhage. In one rat the pancreas was oedematous and in another had a dead-white opaque appearance.

Rats on 100 ppm. DMN lived 9–14 weeks. One of the six had haemorrhage into the gut. The livers were smaller than normal but irregular in shape and some had " fatty " lobes. In several the pancreas was either oedematous or prominently white and opaque. Rats on 50 ppm. killed after 20 weeks in this first experiment showed no gross abnormality except a general rounding of the edges of the liver.

*Histology.*—In those rats dying after 5 weeks on 200 ppm. DMN, the liver was intersected by irregularly-shaped bands of tissue composed of hepatic cells in various degrees of degeneration, red cells, macrophages, with and without yellow pigment, fibroblasts and fine eosinophil fibrils (Fig. 1). Silver impregnation preparations showed increase and condensation of reticulin in the strands (Fig. 2). The parenchymal cells showed more large nuclei and binucleate forms than usual, but mitotic figures were very rare. Very early bile-duct proliferation was occasionally seen.

Rats dying after 9 weeks on a diet with 100 ppm. DMN had rather similar liver lesions with more fibrous tissue (Fig. 3). The parenchymal cells showed similar cytological changes, and were occasionally grouped into nodules surrounded by very fine fibrous capsules. Bile-duct proliferation was well established (Fig. 4).

In those dying later (13 weeks) bile-duct proliferation was rather more advanced and the new ducts were moderately dilated, with some flattening of the epithelium. These structures now had an obvious resemblance to the huge forms to be described later. Occasional hyperplastic parenchymal nodules occurred (Fig. 5) and there was evidence of diffuse hyperplasia of the parenchymal cells. The hyperplastic liver cells often showed very large nuclei with large, often multiple nucleoli. The term hyperplasia is used here in a purely descriptive sense, following Opie (1944) who defined it as being " characterised by changes in the size, staining, and arrangement of cells, and may occur diffusely with no sharp demarcation or in foci, so that well defined nodules are formed ".

The rats on 50 ppm. DMN killed after 20 weeks had livers which showed much less general damage. The main difference from the normal was cytological. There was a generalised increase in cell size, with large nuclei and nucleoli. In one animal well established bile-duct proliferation was present.

*Second experiment*

Ten male and 10 female rats were given a diet containing 50 ppm. DMN with 5 males and 5 females as controls on the same diet with arachis oil alone. The rats on DMN did not grow as well as their controls and their average weight, together with the mean weekly food intake for the 1st, 13th and 26th weeks are given in Table I. Up to this time rats had appeared well, but during the 27th week the first death took place. Deaths continued at intervals until the 42nd week, when the single survivor was killed. One male control rat died with a pulmonary abscess in the 6th week. The rest were healthy when killed at the end of the experiment. One was found to have a sarcoma of the mesenteric glands at autopsy. Of the 20 animals receiving DMN 11 died from effects of an acute haemorrhage into the peritoneal cavity. Bleeding had taken place from the surface of the liver at a site usually marked by adherent omentum. In every animal the liver showed a gross irregularity of its surface with a varying number of nodules scattered throughout. Damage was often more severe on the left side, where the lobes were shrunken and distorted to a marked extent. The irregular nodular appearance of the right side of the liver is shown in Fig. 6 while one of the smaller left lobes is distorted by extravasated blood. A normal liver (Fig. 7) is shown for comparison. Numerous small translucent cysts were frequently seen in addition to the solid nodules. The most striking feature of these livers was the irregularity in outline and variation in the shape of individual lobes (Fig. 8). Uniformly shrunken fibrotic livers were not seen.

TABLE I.—*Mean Weight with Standard Error and Weekly Food Intake in g. per* 100 *g. Rat for Rats on a Diet Containing* 50 *ppm. DMN.* (*Number of rats in brackets.*)

| | | Start. | 13 weeks. | 26 weeks. |
|---|---|---|---|---|
| *Male :* | | | | |
| Control (4) | Body weight | 111 | 293 ± 27 | 351 ± 17 |
| | Food intake | 78 | 48·5 | 45 |
| DMN (10) | Body weight | 103 | 245 ± 21 | 293 ± 32 |
| | Food intake | 74·5 | 47 | 37 |
| *Female :* | | | | |
| Control (5) | Body weight | 104 | 212 ± 16 | 244 ± 18 |
| | Food intake | 67·5 | 49·5 | 49 |
| DMN (10) | Body weight | 110 | 184 ± 22 | 209 ± 20 |
| | Food intake | 71 | 52·5 | 48 |

In those animals dying later in the experiment there was usually a mass of red lymph nodes in the portal fissure. The pancreas in two rats had an opaque dead-white appearance, but otherwise the abdominal viscera appeared normal. In the chest the mediastinal glands were usually red and the lungs showed either a general congestion or more commonly, a number of red petechiae scattered over the pleural surface. Pleural exudates were not encountered.

Of the 9 animals not dying from an acute internal haemorrhage, one rat, the first to die, had a peritoneal cavity filled with orange coloured exudate, and the liver distorted by a large rubbery mass on its proximal surface resembling a sarcoma. No primary tumour could be found elsewhere. Of the other 8 rats, one only was killed in good condition as the sole survivor after 40 weeks. Its liver contained several nodules. The others were killed because they were ill. All had nodular livers and in three omentum was adherent to parts of the liver with indications of a small haemorrhage from the liver surface. Another had a peritoneal cavity full of clear fluid and two had serious involvement of the lungs. No rat appeared to have died solely from liver failure due to necrosis or destruction of the organ by fibrosis.

Male and female rats appeared to respond equally to DMN with the same mean survival time and similar lesions in their livers.

*Histology.*—With one exception the animals killed or dying at different times during the experiment showed liver lesions of a similar character, therefore a general description of the histological changes will be given.

In harmony with the gross appearance there was great variation in the histological picture seen in sections taken from different parts of the same liver. The more grossly damaged areas showed complete absence of the normal architecture and were hardly recognisable as hepatic (Fig. 9).

Parenchymal nodules were seen in every liver, many being similar to those described in the first experiment. They were roughly circular, surrounded by a fibrous capsule, and appeared to be compressing the surrounding structures (Fig. 10 and 11). They were composed of closely packed parenchymal cells, which lacked the normal trabecular arrangement, and there was great variation in cell and nuclear size, but mitotic figures were infrequent. Occasional small bile ducts and fairly frequent sinusoids were seen. Some of the nodules showed necrosis and haemorrhage in their central parts. In the extreme cases almost the whole structure was replaced by blood clot, which was sometimes undergoing organisation. Diffuse parenchymal hyperplasia was also present. No sharp distinction could be drawn between advanced hyperplasia and true neoplasia of the parenchymal cells. Changes characteristic of neoplasia were considered to be the presence of cells varying extremely in size and shape with bizarre multinucleated forms. These cells had large nuclei with either a single large or multiple deeply basophilic nucleoli. Mitotic figures were common and frequently abnormal (Fig. 12). Neoplastic change of this type was seen in areas of hyperplasia, most frequently in the nodules. Typically, the lesion consisted of an outer zone of neoplastic cells surrounding a central haemorrhagic necrotic mass mass (Fig. 13). This appearance was also typical of that seen in metastatic tumours (Fig. 14). The tumours were mainly anaplastic with varying degrees of pseudo-acinar formation.

Bile-duct proliferation was always present and showed much variation in the form of the proliferated ducts and in the amount of the accompanying fibrous tissue. The most frequent appearance was of large multilocular cystic structures, which corresponded to the yellow translucent cysts seen in the gross specimens. They were lined by a thin layer of flattened epithelium with only a sparse fibrous framework between the loculi, and with little or no surrounding fibrosis (Fig. 15). In other areas the cystic dilatation was less marked or absent and the epithelium cubical or low columnar. Here the interstitial and surrounding fibrous tissue was more abundant. No continuity with normal bile-ducts was seen, but the presence of a wide range of intermediate forms together with the appearance of the lining epithelium suggested a common biliary origin for the lesions (serial sections were not made). In some places, the structural irregularity was very marked and the epithelial nuclei were hyperchromatic (Fig. 16), but mitotic figures were rare and this type of lesion was not found in the metastases.

A third type of lesion seen in only one animal had the appearance of a fibrosarcoma. The tumour mass consisted of large numbers of fibroblasts, many abnormal, with frequent mitotic figures. Preparations stained by Mallory's method showed much collagen production (Fig. 17). There were occasional tubular structures within the tumour resembling bile ducts. No metastases were found in this animal, but the tumour was locally invasive.

Intravascular ante-mortem thrombosis was occasionally found (Fig. 18), the type of vessel being difficult to identify because of the gross general distortion of the liver. Some fibrosis was present in all the livers, but this was never a very prominent feature and again no precise anatomical distribution could be assigned.

Nineteen out of the 20 rats developed primary hepatic tumours. Metastases were present in 7 of the animals, 4 being pulmonary and the remainder intra-abdominal. No histological evidence of primary neoplasia was found except in the liver. The sole surviving rat, which was killed in apparent good health, showed slight diffuse and nodular hyperplastic change in its liver parenchyma.

*Third experiment*

Six male rabbits (2·1–2·8 kg.) were given a diet containing 20 ppm. DMN. A lower level was used because rabbits are more sensitive than rats to the acute effects of DMN. After 10 weeks five of the rabbits had lost a little weight, but otherwise appeared normal, the sixth had lost more weight and appeared ill. The concentration of DMN was raised to 30 ppm. for another 4 weeks and to 50 ppm. for a further 8 weeks, by which time all the rabbits had died. One was killed during the 11th week. Four others became ill during the 18th–20th weeks and were killed and the last animal was killed in the 22nd week. They showed no signs of poisoning beyond a gradual loss of weight and increased listlessness and general weakness. In every case the liver was small (25–35 g.) dark and slightly, but not grossly, fibrosed, as judged by the ease with which it was cut. Nodules, cysts and haemorrhages were not seen. The other abdominal organs appeared normal and the loss of condition of the animals could only be attributed to a progressive loss of liver tissue.

*Histology.*—No evidence of neoplasia was found in the livers which showed occasional areas of early bile-duct proliferation and parenchymal hyperplasia, also some slight irregular fibrosis.

### DISCUSSION

As far as we are aware, the experimental production of liver tumours by dimethylnitrosamine has not been reported previously, therefore our findings will be discussed in relation to those of other workers using different agents.

Several substances have been shown to produce primary liver tumours after repeated small doses, and there is a definite similarity in the character of the lesions produced. Thus some of the compounds when administered in single larger doses give rise to an acute centrilobular type of necrosis involving all lobules, followed by evidence of parenchymal cell regeneration. Butter yellow (Orr and Price, 1948); carbon tetrachloride (Cameron and Karunaratne, 1936); thioacetamide (Ambrose, De Eds and Rather, 1949; Gupta, 1955) and senecio alkaloids (Davidson, 1935) all have this property and DMN falls into the same group. It must be emphasized that with the exception of senecio, DMN causes a much greater amount of haemorrhage associated with the necrosis than the other compounds.

Chronic choline deficiency (Salmon and Copeland, 1954) and feeding with bentonite, which is believed to induce the same condition (Wilson, 1954) on the other hand do not produce centrilobular necrosis, but diffuse fatty change which is followed by fibrosis and tumour formation. In chronic ethionine feeding (Popper, de la Huerga and Koch-Weser, 1954), fatty change and central necrosis precede lesions which are probably neoplastic.

It appears, therefore, that the behaviour of DMN is comparable to that of the tumour-producing agents which are able to cause acute centrilobular necrosis. The early morphological changes in the liver, as shown by the rats receiving 200 ppm. for 5 weeks, are consistent with such a comparison. The formation of strands of tissue destruction with macrophage infiltration and early fibrosis (Fig. 1) is similar to that described by Orr (1940) using butter yellow. At the same time many of the parenchymal cells showed increased size, including enlargement of the nuclei and nucleoli, and there was early but recognisable bile-duct hyperplasia. Similar changes in parenchymal cell size have been noted by Ambrose, De Eds and Rather (1949) using thioacetamide, and have been the subject of special investigations by Rather (1951) and Kleinfeld and Lessler (1954) who agree that they are the earliest morphological abnormality. In the present work no animals were studied earlier than five weeks after the start of DMN administration, therefore no opinion can be expressed on the exact chronology of the appearance of the lesions.

The rats on 50 ppm. DMN which were examined before the 26th week were from the first experiment and they showed minimal liver changes except one with characteristic liver damage. The rats in both experiments were from the same stock, but the first experiment was carried out during the winter and the second started in the summer.

In discussing the tumours, problems of nomenclature arise. The term hepatoma is defined in Blakiston's *New Gould Medical Dictionary* (1949) as " Any tumour originating in the liver ; applied more particularly to nodular foci of regeneration, to adenomas, and to that form of primary hepatic carcinoma made up of cells which, in arrangement and form, resemble the cells of the hepatic cords ". Both the restricted and the general sense of the word have been used by writers on experimental hepatic tumours. A further difficulty is created by nomenclature implying pathogenesis, thus such terms as bile-duct carcinoma and cholangioma indicate the origin of the neoplasm in biliary epithelium, while hepatoma may imply that it arises in hepatic cells. In the case of tumours induced by butter yellow, there is considerable disagreement on the cell of origin. Opie (1944) ; Orr (1940) ; Cortell (1947) and Richardson and Borsos-Nachtnebel (1951) maintain that tumours can arise from both hepatic cells and biliary cells. Edwards and White (1941) and Dalton and Edwards (1942), however, believe that all true neoplasia in the liver due to butter yellow has its origin in the hepatic cell only, and that changes in the bile-ducts are to be interpreted as hyperplastic. A third group of workers (Price, Harman, Miller and Miller, 1952) working with derivatives of butter yellow have concluded that most, if not all, of the neoplasms, regardless of their histological pattern, arise from areas of cholangiofibrosis and thus have a common pathogenesis.

As far as possible, therefore, in the present paper terms which imply a definite pathogenesis have been avoided.

The distinction between advanced hyperplasia and true neoplasia must ultimately be subjective, but the existence of metastatic spread provides objective evidence of malignancy. As Opie (1944) has pointed out " histological structure is an uncertain index of malignancy, tumours with metastases have been regarded as decisively malignant, they reproduce these distinctive characteristics in the metastases that formed ". We have made no attempt to distinguish between advanced hyperplasia and benign neoplasia, but in some instances a lesion has been regarded as malignant in the absence of observed metastasis, if its histological and cytological structure was indistinguishable from that found in tumours with definite evidence of distant spread. A significant number of tumours did in fact show metastases, and the histological and cytological characteristics of the secondary tumours have been regarded as criteria for the diagnosis of neoplasia in the lesions occurring in the liver. With these criteria in mind it can be said that the tumours caused by DMN are predominantly anaplastic with some tendency to the formation of structures resembling glands. The difficulties of assigning a cell of origin have been discussed above. However, these tumours appear to arise either in hyperplastic parenchymal nodules or in regions of diffuse

---

EXPLANATION OF PLATES.

FIG. 1.—Rat Liver. First experiment. DMN 200 ppm. for 5 weeks. Shows strands of damaged tissue described in the text. H. & E. × 35.

FIG. 2.—Rat Liver. First experiment. DMN 200 ppm. for 5 weeks. Shows reticulin in the strands. Silver impregnation. × 70.

FIG. 3.—Rat Liver. First experiment. DMN 100 ppm. for 9 weeks. Shows the formation of fibrous tissue. Mallory. × 70.

FIG. 4.—Rat Liver. First experiment. DMN 100 ppm. for 9 weeks. Shows early bile-duct proliferation. H. & E. × 70.

FIG. 5.—Rat Liver. First experiment. DMN 100 ppm. for 13 weeks. Shows a hyperplastic nodule. H. & E. × 70.

FIG. 6.—Rat Liver. Second experiment. DMN 50 ppm. Shows the irregular nodular appearance of the liver, with a lobe distorted by extravasated blood. The scale is graduated in centimetres.

FIG. 7.—Rat Liver. Second experiment. Control liver for comparison.

FIG. 8.—Rat Liver. Second experiment. To show irregularity in outline and variation in shape of individual lobes.

FIG. 9.—Rat Liver. Second experiment. To show the extremely severe structural change in the liver. H. & E. × 3.

FIG. 10.—Rat Liver. Second experiment. A typical hyperplastic nodule. H. & E. × 60.

FIG. 11.—Rat Liver. Second experiment. The same nodule as Fig. 10, Mallory. × 75.

FIG. 12.—Rat Liver. Second experiment. Part of a tumour under higher magnification showing extreme anaplasia, huge nucleoli and a large abnormal mitotic figure. H. & E. × 300.

FIG. 13.—Rat Liver. Second experiment. Typical tumour consisting of a rim of neoplastic tissue surrounding a massive central core of necrotic debris and blood clot. H. & E. × 3.

FIG. 14.—Rat Lung. Second experiment. Shows a pulmonary metastasis. H. & E. × 30.

FIG. 15.—Rat Liver. Second experiment. Shows very severe cystic bile-duct proliferation. H. & E. × 43.

FIG. 16.—Rat Liver. Second experiment. Very irregular bile-duct hyperplasia with hyperchromatic nuclei. H. & E. × 85.

FIG. 17.—Rat Liver. Second experiment. Sarcoma, to show the general structure and production of collagen. Mallory connective tissue method. × 85.

FIG. 18.—Rat Liver. Second experiment. Showing ante-mortem intravascular thrombosis. H. & E. × 85.

1

2

3

4

5

6

7

8

9

10

Magee and Barnes.

11

12

14

13

Magee and Barnes.

15

16

17

18

Magee and Barnes.

parenchymal hyperplasia and their cytology shows more resemblance to hepatic than to biliary cells. The invariable accompaniment of massive necrosis and haemorrhage increases the difficulty of exact classification.

The enormous cystic structures so commonly observed are regarded as extreme forms of bile-duct proliferation, as all intermediate degrees from normal ducts can be found. They appear to correspond to the cystadenomata of Orr (1940) and Opie (1944). The neoplastic character of this type of lesion has been contested by Edwards and White (1941). Occasionally, in areas of duct proliferation, the structure was much more irregular and the cells contained larger and hyperchromatic nuclei. Mitotic figures were not common however, and this type of structure was never seen in metastatic growths, therefore its classification remains in doubt.

The single sarcomatous tumour remains. Here again no metastasis was found, but the invasive character and the frequent mitotic figures suggested malignancy. More important is the question whether this tumour was caused by DMN or whether it arose spontaneously. No similar tumour was observed in the livers of control animals, nor indeed in any rat in our colony. Richardson and Borsos-Nachtnebel (1951) have noted fibrosarcoma and angiosarcoma in rats fed 3-methyl-4-dimethylamino azobenzene, and Firminger and Mulay (1952) described apparent sarcomatous change in the stroma of an adenocarcinoma induced by azo dye.

As already emphasized, haemorrhage and necrosis are constant and prominent features in acute and chronic DMN poisoning. Ischaemic factors played a part in the production of necrosis, notably in the hyperplastic nodules. A disordered vascular system has been shown to be present in malignant neoplasms of the liver (including butter yellow tumours in rats) by Breedis and Young (1949), who state that these tumours tend to acquire an exclusively arterial blood supply.

Apart from its intrinsic interest, a knowledge of the cell of origin in experimental hepatic tumours is of some importance in relation to chemical investigation. Mueller and Miller (1953) have shown that butter yellow and certain of its derivatives are metabolised *in vivo* and *in vitro*, and unpublished work in our laboratory indicated that DMN also undergoes rapid metabolic alteration. These metabolic reactions may be concerned in the carcinogenic process, and there may be some correlation between the cells which mediate them and those which ultimately become neoplastic.

DMN may prove a useful agent in the study of experimental hepatic carcinogenesis. It is miscible with water in all proportions and can be readily estimated in tissues by a simple polarographic method (Heath and Jarvis, 1955).

At present no hypothesis can be advanced on the mechanism of action of DMN in the production of tumours. It may be noted that both DMN and butter yellow have N-dimethyl groups.

$(CH_3)_2NNO$

Dimethylnitrosamine p-dimethyl-amino-azobenzene
(butter yellow)

It has been shown that at least one N-methyl group is required for the carcinogenic activity of dyes related to butter yellow in the liver of the rat (Miller and Miller, 1953).

### SUMMARY

Twenty rats, divided into two groups according to sex, were fed a normal diet (M.R.C. diet 41) to which had been added dimethylnitrosamine at a level of 50 parts per million. Between the 26th and 40th week of this treatment 19 of the animals developed primary hepatic tumours, metastatic spread being present in 7 cases. An attempt to produce tumours in rabbits by the same agent was unsuccessful. Dimethylnitrosamine, by virtue of its chemical and physical properties, may be of some value in the investigation of hepatic carcinogenesis.

We would like to thank Professor G. R. Cameron, F.R.S., for very valuable discussion and criticism. Mr. R. Legg, A.I.M.L.T., made the histological preparations and photomicrographs.

### REFERENCES

AMBROSE, A. M., DE EDS, F. AND RATHER, L. J.—(1949) *J. industr. Hyg.*, **31**, 158.
BARNES, J. M. AND MAGEE, P. N.—(1954) *Brit. J. Industr. Med.*, **11**, 167.
BLAKISTON'S *NEW GOULD MEDICAL DICTIONARY*—(1949) Philadelphia and Toronto (The Blakiston Company).
BREEDIS, C. AND YOUNG, G.—(1949) *Fed. Proc.*, **8**, 351.
BRUCE, H. M.—(1947) *J. Hyg., Camb.*, **45**, 169.
*Idem* AND PARKES, A. S.—(1949) *Ibid.*, **47**, 202.
CAMERON, G. R. AND KARUNARATNE, W. A. E.—(1936) *J. Path. Bact.*, **42**, 1.
CORTELL, R.—(1947) *Cancer Res.*, **7**, 158.
DALTON, A. J. AND EDWARDS, J. E.—(1942) *J. nat. Cancer Inst.*, **3**, 319.
DAVIDSON, J.—(1935) *J. Path. Bact.*, **40**, 285.
EDWARDS, J. E. AND WHITE, J.—(1941) *J. nat. Cancer Inst.*, **2**, 157.
FIRMINGER, H. I. AND MULAY, A. S.—(1952) *Ibid.*, **13**, 19.
GUPTA, D. N.—(1955) *Nature*, **175**, 257.
HEATH, D. F. AND JARVIS, J. A. E.—(1955) *Analyst*, **80**, 613.
KLEINFELD, R. G. AND LESSLER, M. A.—(1954) *Amer. J. Physiol.*, **179**, 651.
MILLER, J. A. AND MILLER, E. C.—(1953) *Advances in Cancer Research.* Vol. 1, p. 340. New York, N.Y. (Academic Press Inc.).
MUELLER, G. C. AND MILLER, J. A.—(1953) *J. biol. Chem.*, **202**, 579.
OPIE, E. L.—(1944) *J. exp. Med.*, **80**, 231.
ORR, J. W.—(1940) *J. Path. Bact.*, **50**, 393.
*Idem*, AND PRICE, D. E.—(1948) *Ibid.*, **60**, 461.
POPPER, H., DE LA HUERGA, J. AND KOCH-WESER, D.—(1954) *Ann. N. Y. Acad. Sci.*, **57**, 936.
PRICE, J. M., HARMAN, J. W., MILLER, E. C. AND MILLER, J. A.—(1952) *Cancer Res.*, **12**, 192.
RATHER, L. J.—(1951) *Johns Hopk. Hosp. Bull.*, **88**, 38.
RICHARDSON, M. L. AND BORSOS-NACHTNEBEL, E.—(1951) *Cancer Res.*, **11**, 398.
SALMON, W. D. AND COPELAND, D. H.—(1954) *Ann. N.Y. Acad. Sci.*, **57**, 664.
SCHOENTAL, R., HEAD, M. A. AND PEACOCK, P. R.—(1954) *Brit. J. Cancer*, **8**, 458.
WILSON, J. W.—(1954) *Ann. N.Y. Acad. Sci.*, **57**, 678.


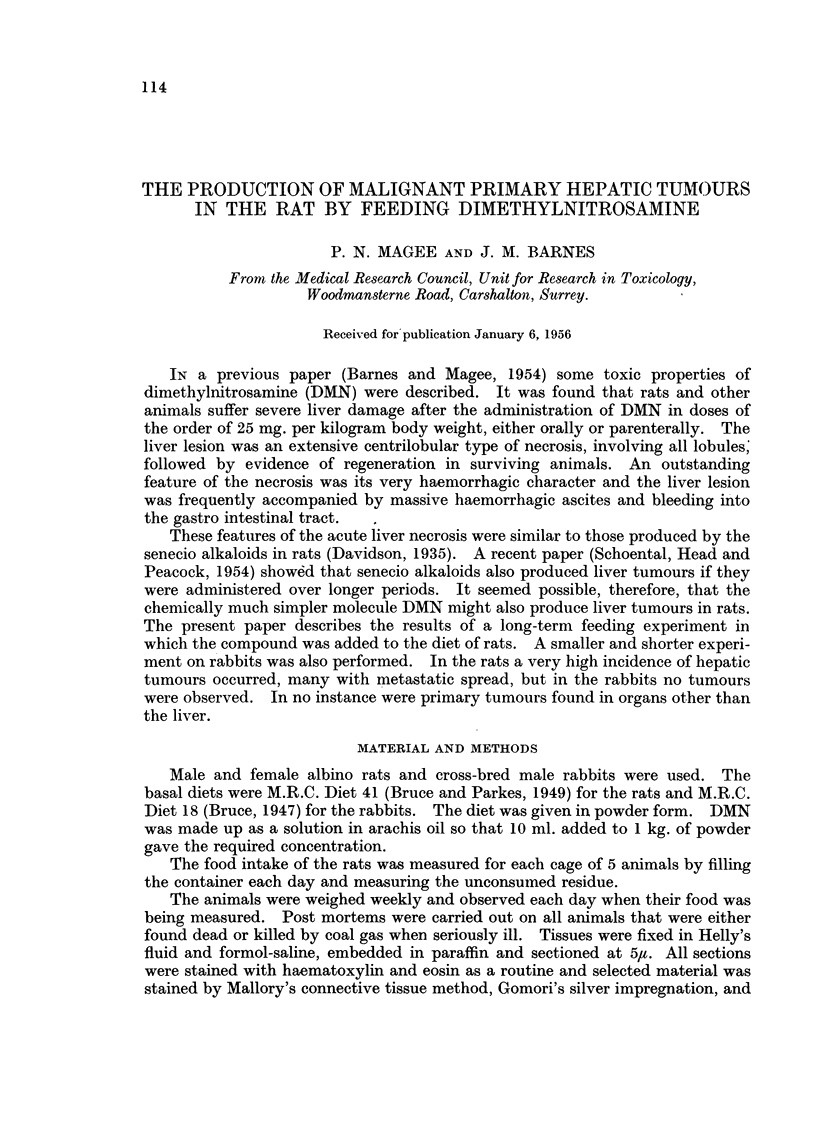

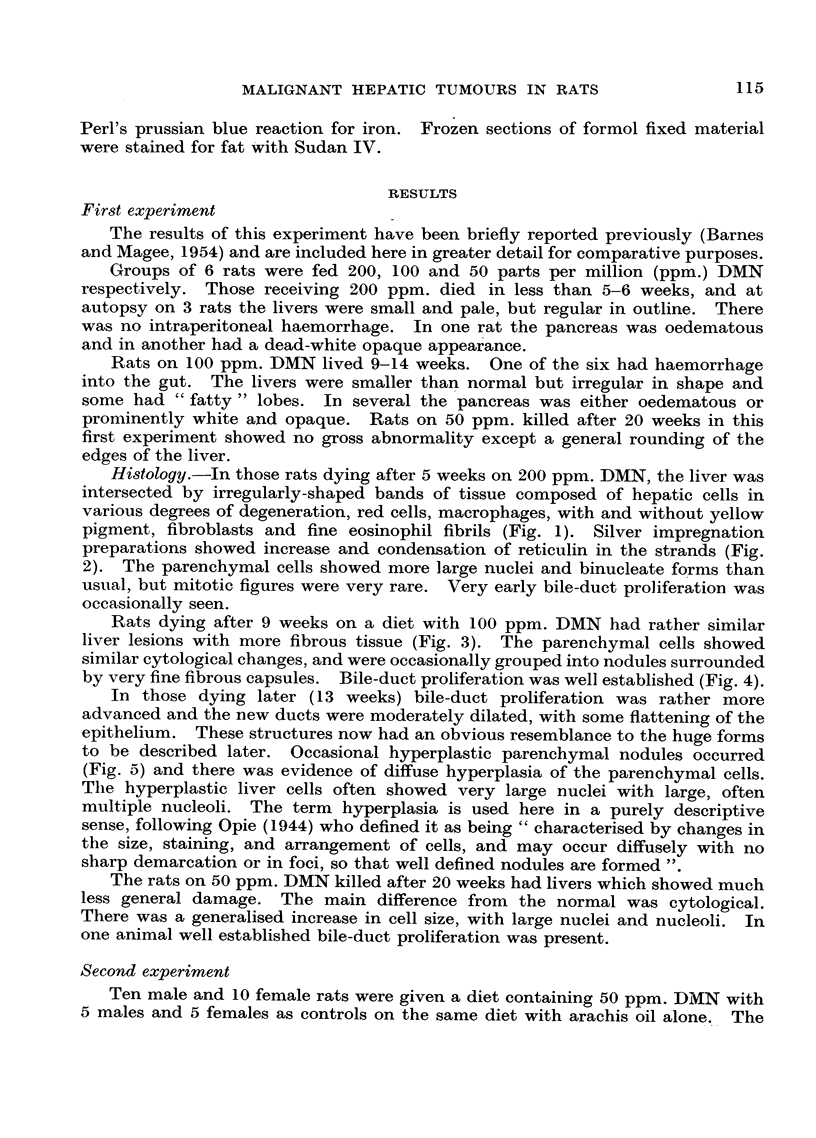

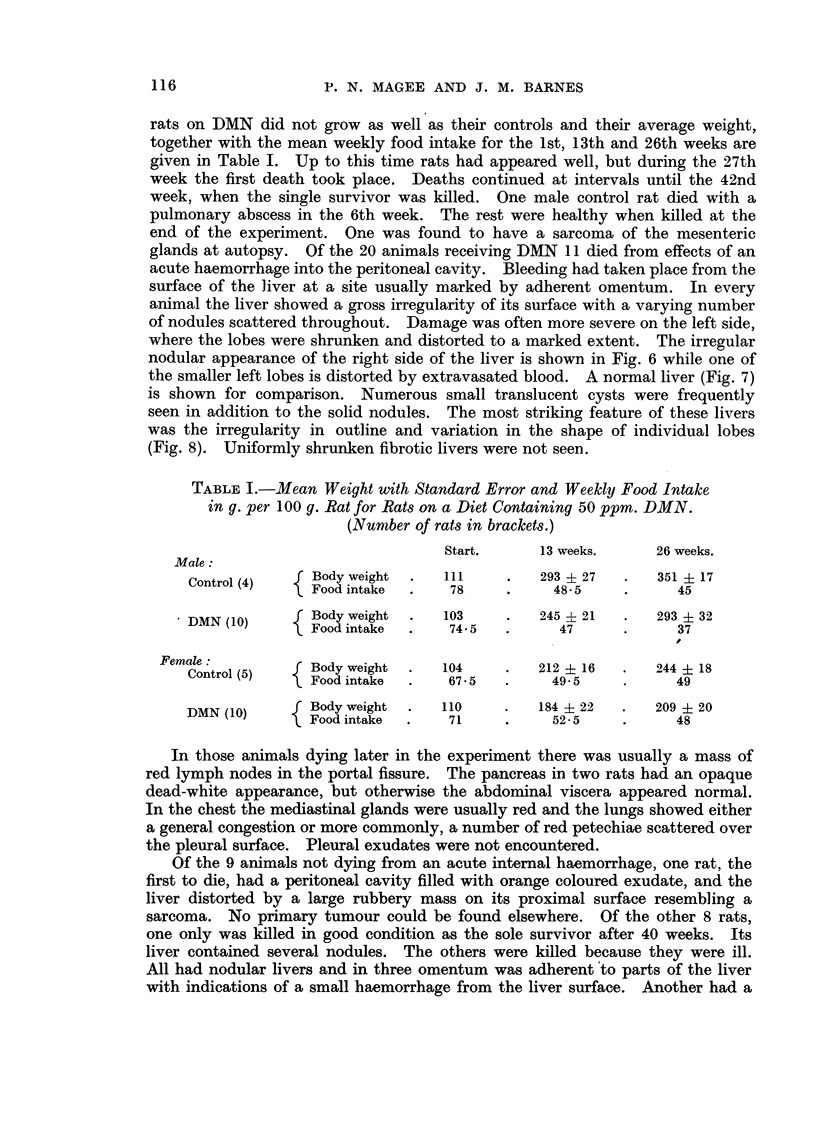

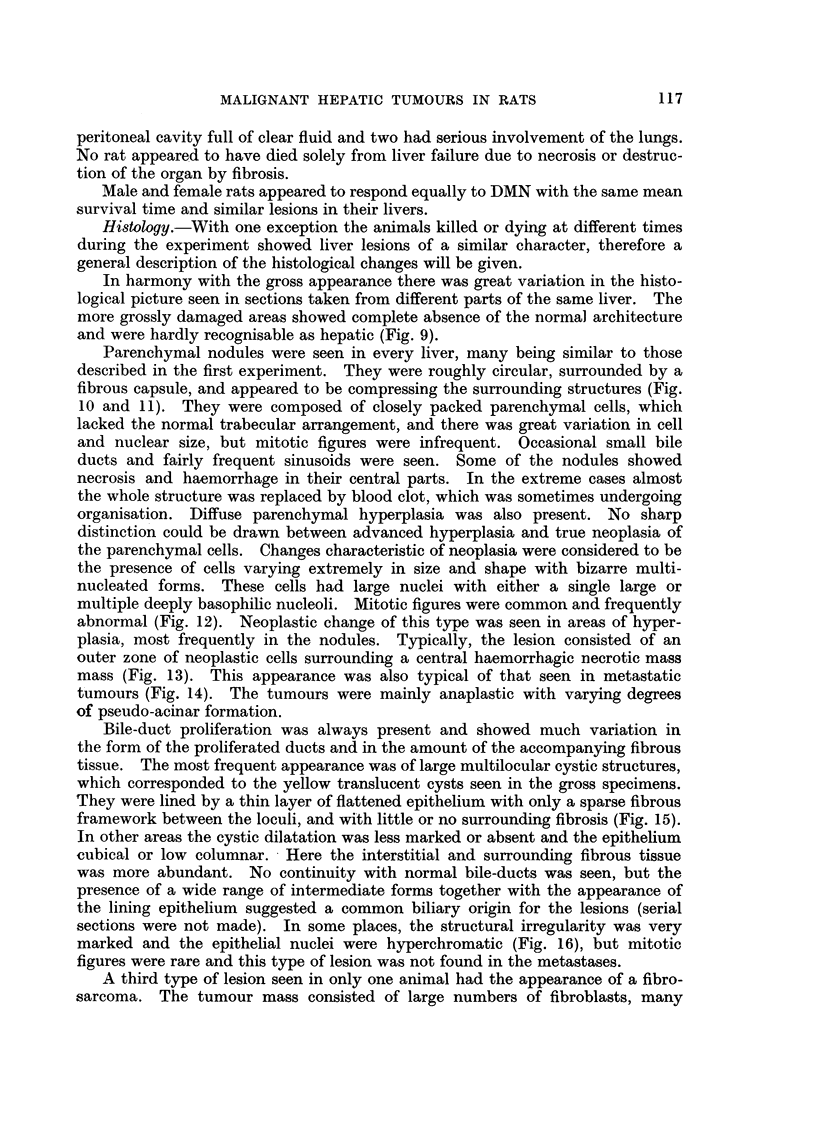

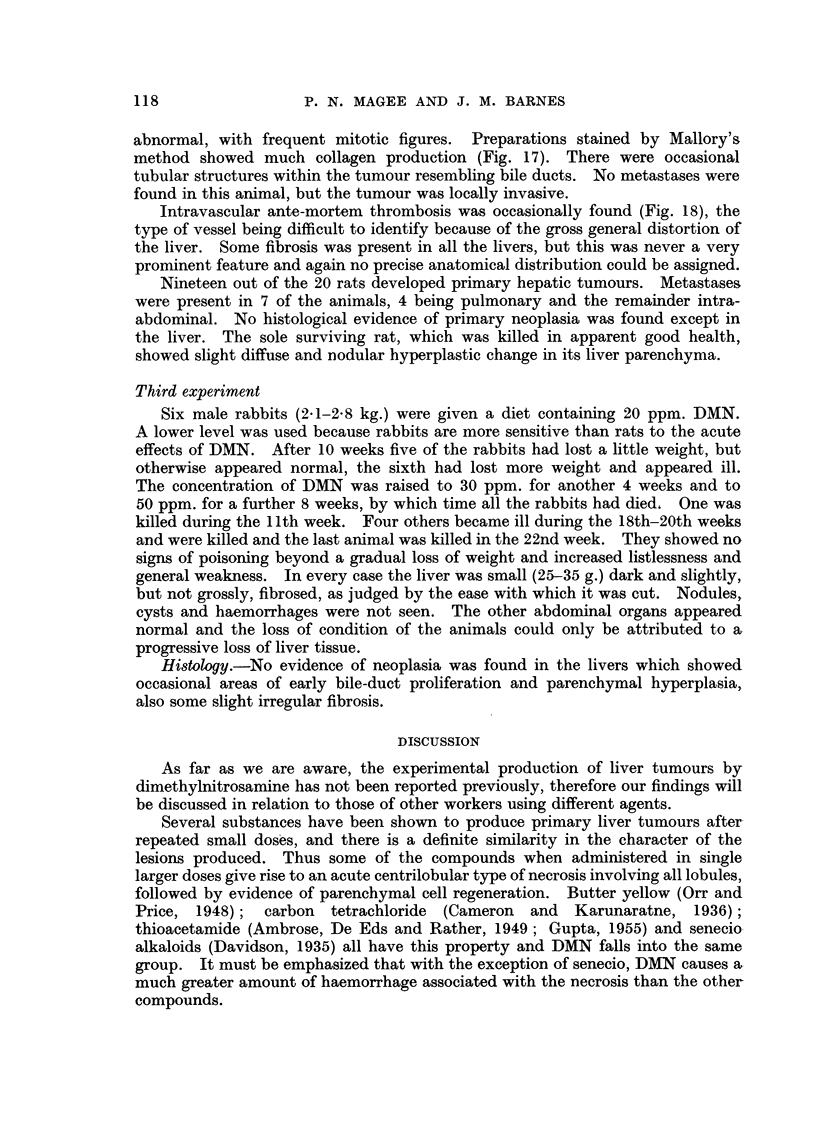

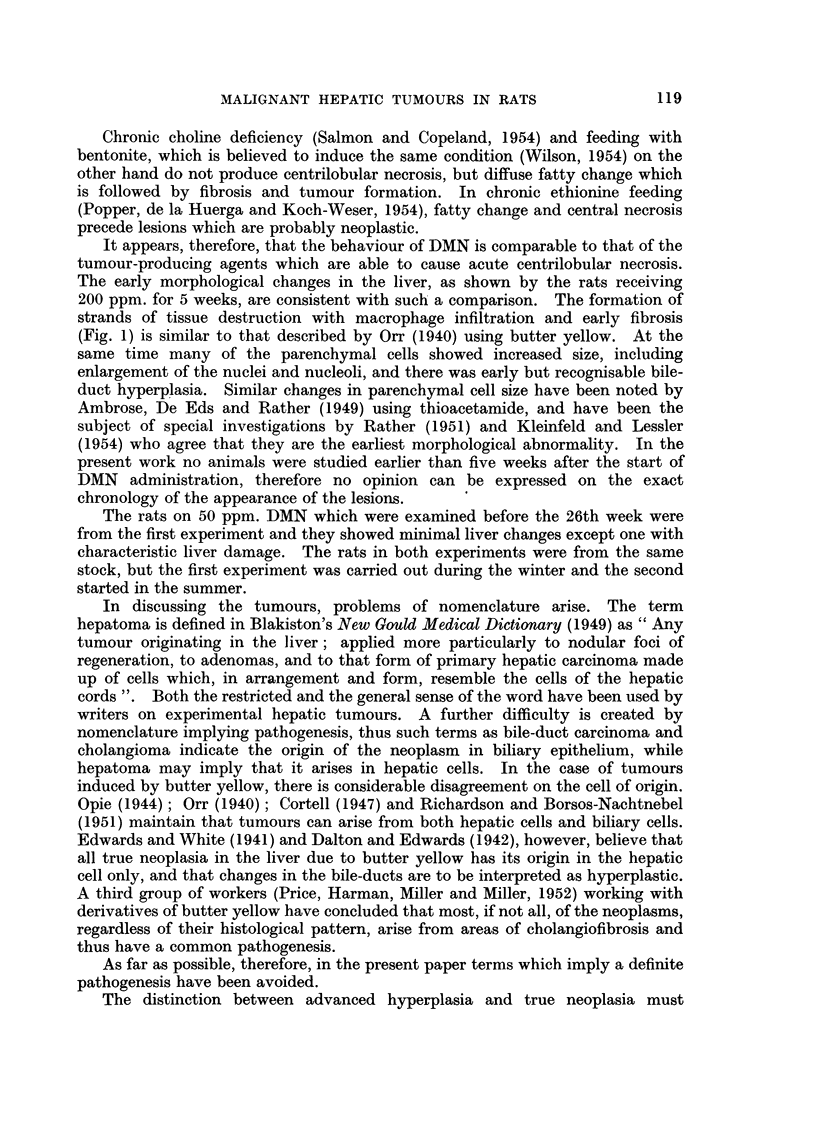

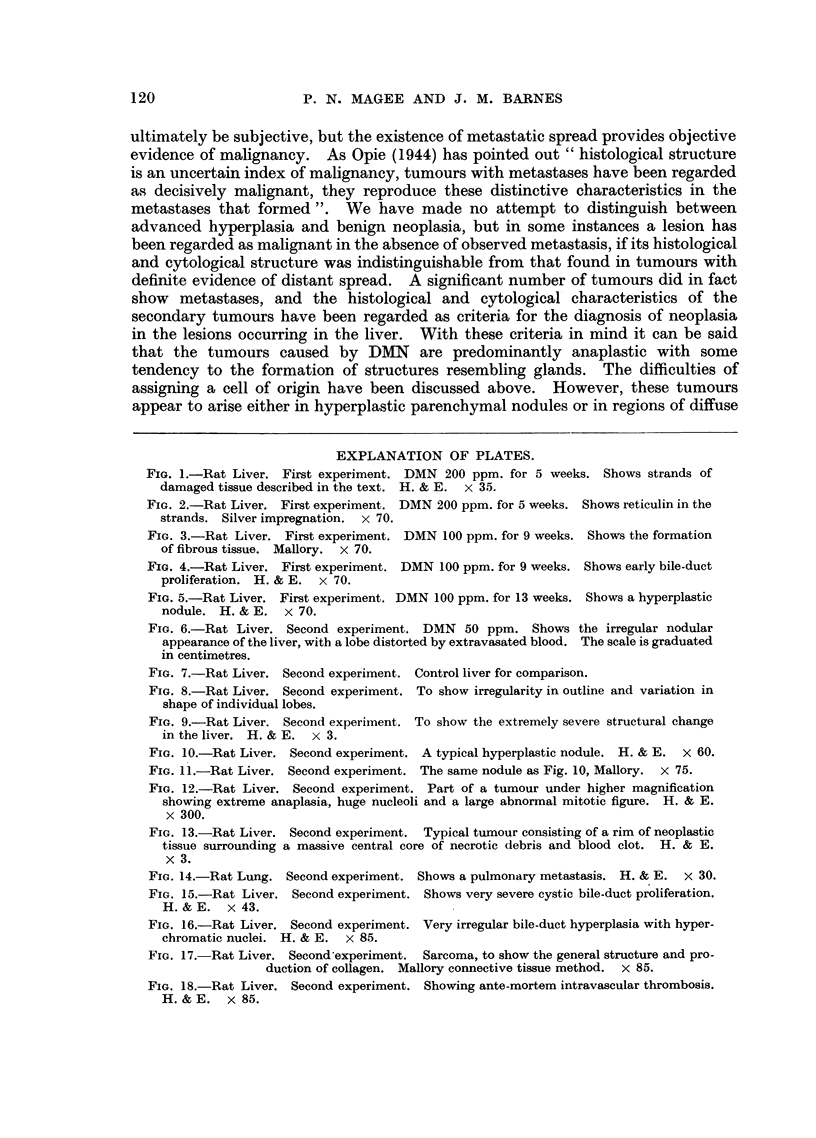

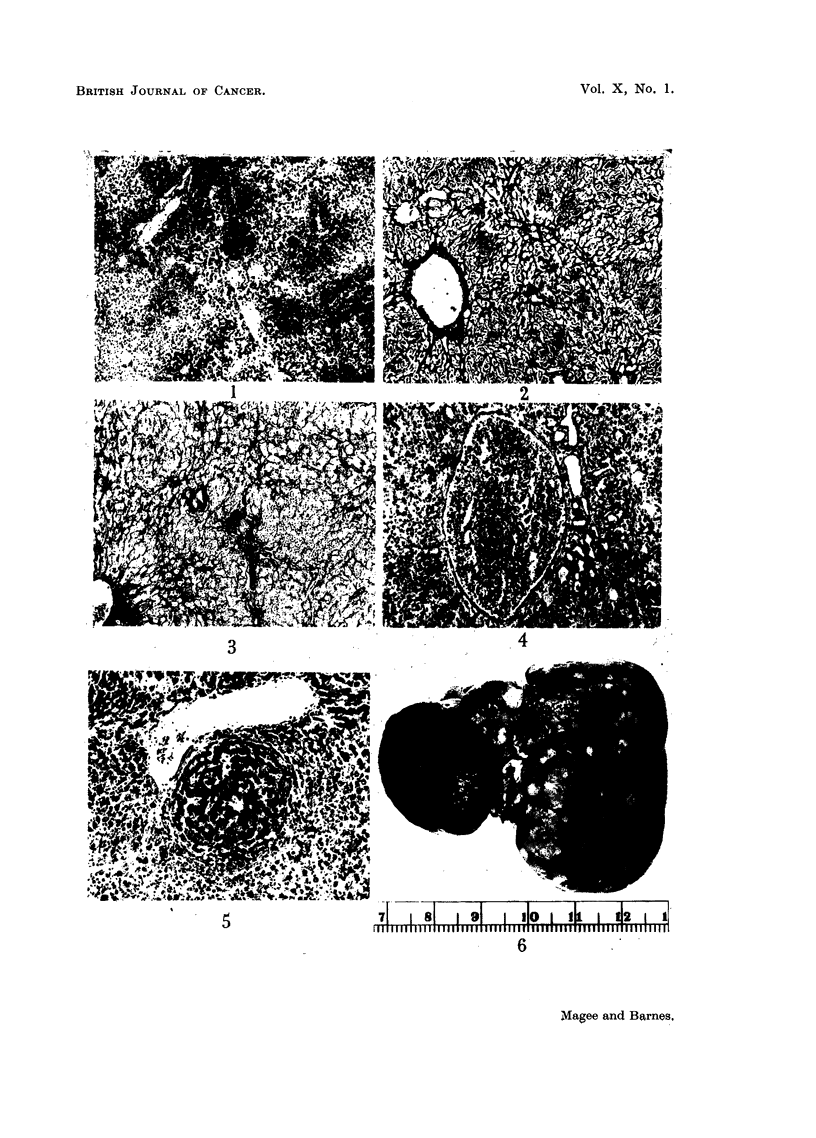

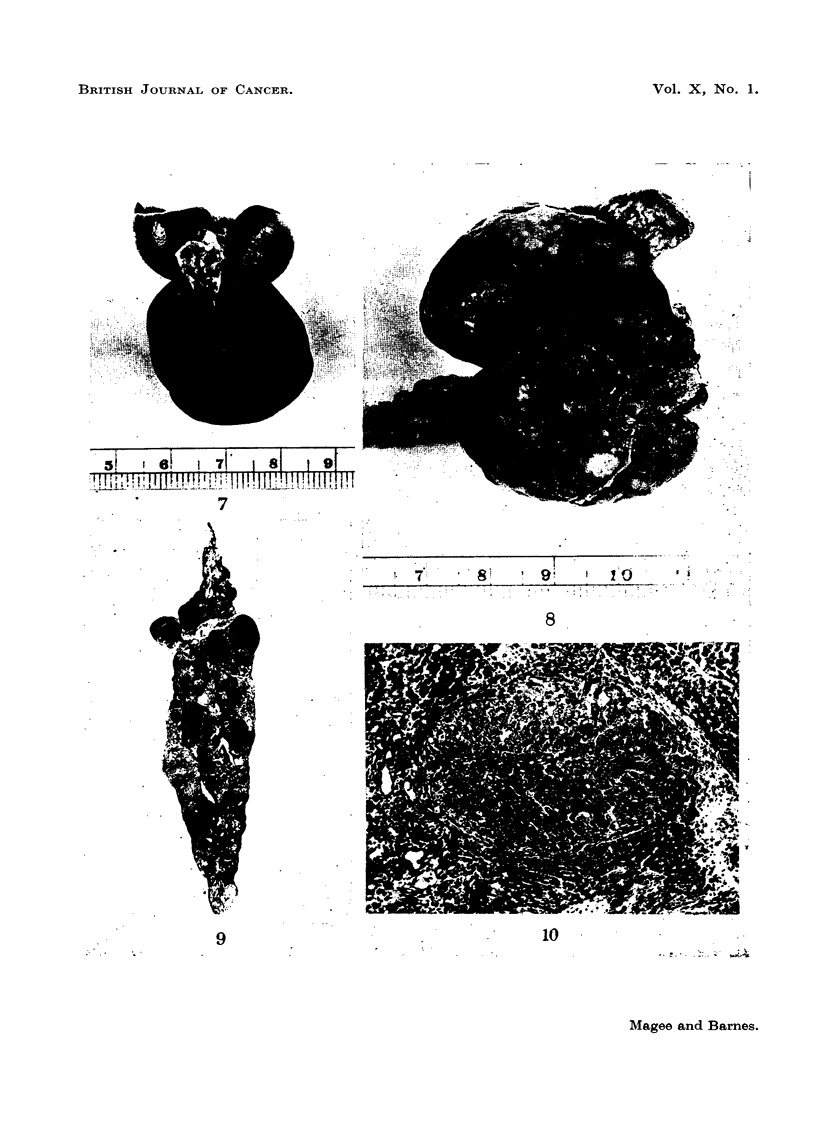

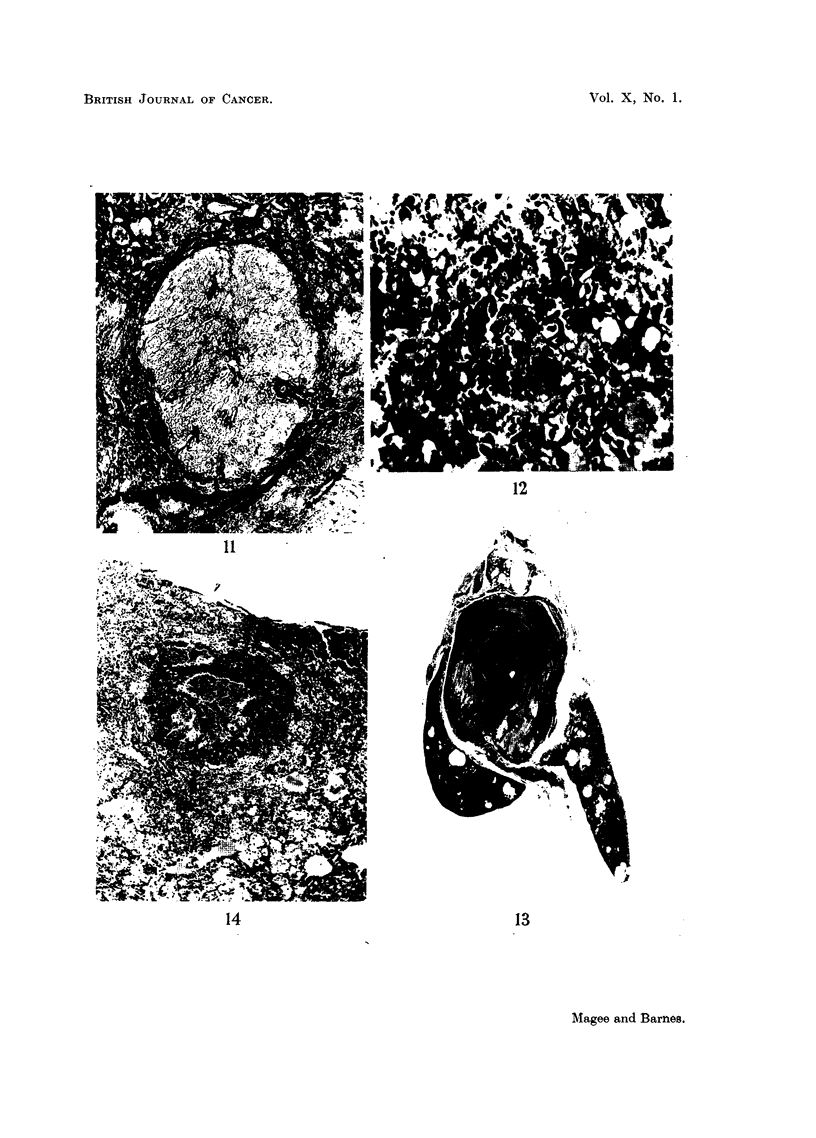

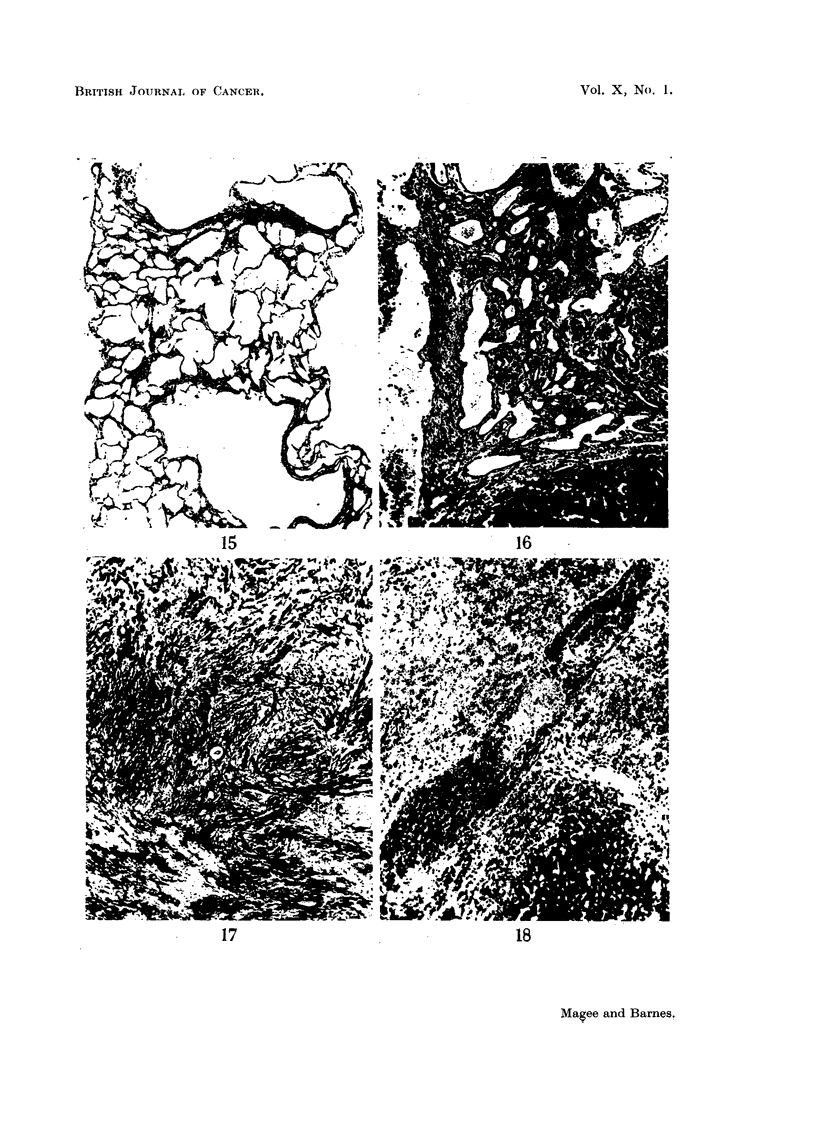

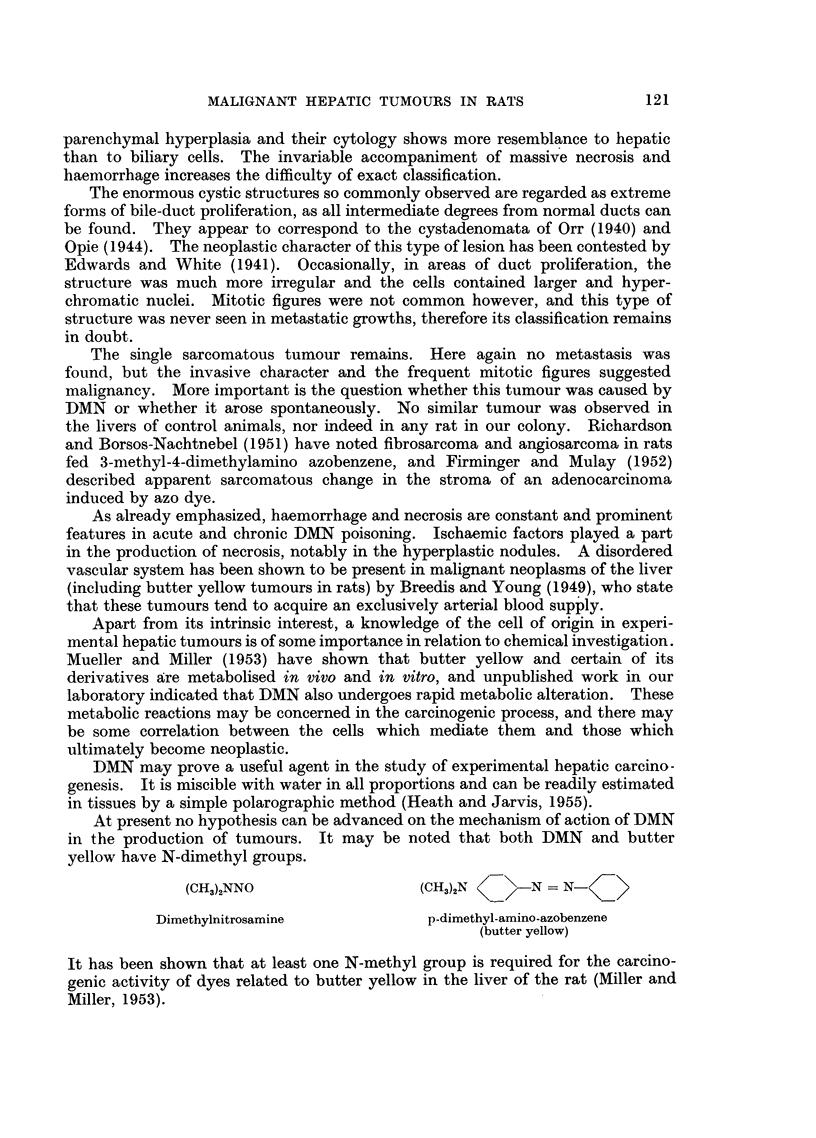

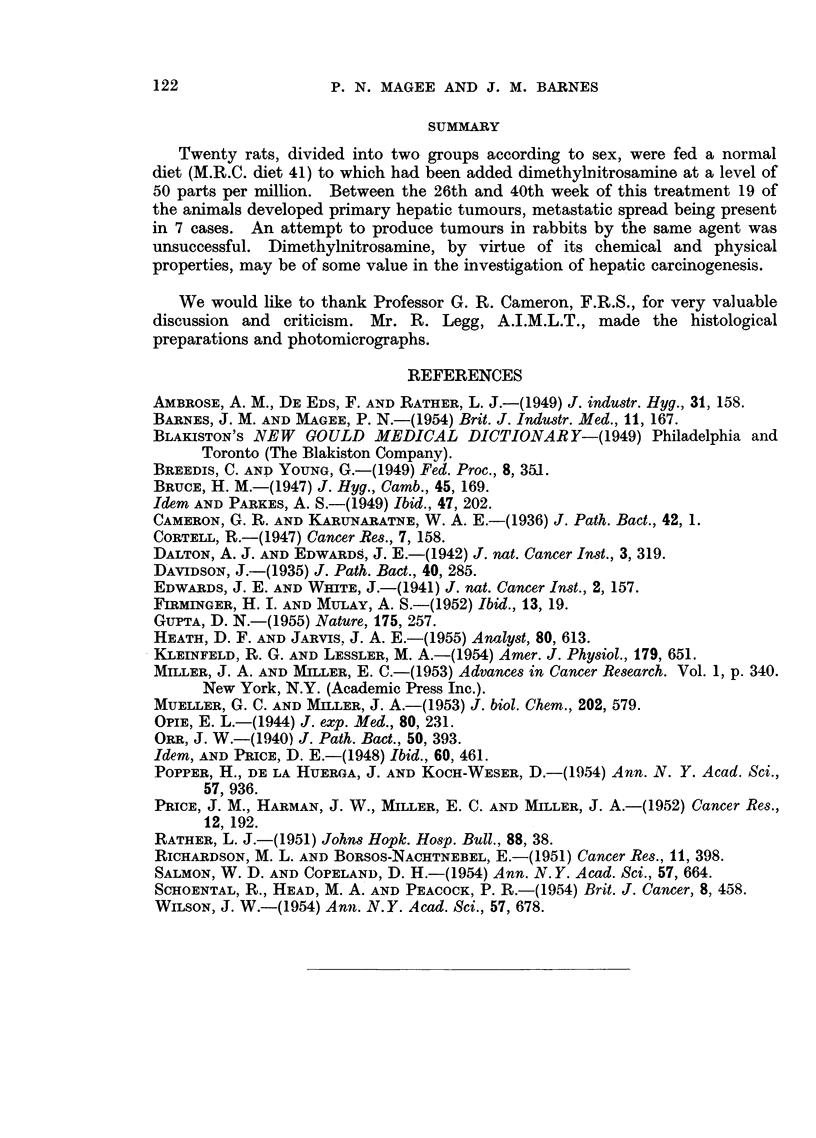


## References

[OCR_00596] BARNES J. M., MAGEE P. N. (1954). Some toxic properties of dimethylnitrosamine.. Br J Ind Med.

[OCR_00604] BRUCE H. M., PARKES A. S. (1949). Feeding and breeding of laboratory animals; a complete cubed diet for mice and rats.. J Hyg (Lond).

[OCR_00616] GUPTA D. N. (1955). Production of cancer of the bile ducts with thioacetamide.. Nature.

[OCR_00625] MUELLER G. C., MILLER J. A. (1953). The metabolism of methylated aminoazo dyes. II. Oxidative demethylation by rat liver homogenates.. J Biol Chem.

[OCR_00631] POPPER H., DE LA HUERGA J., KOCHWESER D. (1954). Hepatic injury due to conditioned sulfo amino acid deficiency.. Ann N Y Acad Sci.

[OCR_00635] PRICE J. M., HARMAN J. W., MILLER E. C., MILLER J. A. (1952). Progressive microscopic alterations in the livers of rats fed the hepatic carcinogens 3'-methyl-4-dimethylaminoazobenzene and 4'-fluoro-4-dimethylaminoazobenzene.. Cancer Res.

[OCR_00641] RICHARDSON H. L., BORSOS-NACHTNEBEL E. (1951). Study of liver tumor development and histologic changes in other organs in rats fed azo dye 3-methyl-4-dimethylaminoazobenzene.. Cancer Res.

[OCR_00644] SCHOENTAL R., HEAD M. A., PEACOCK P. R. (1954). Senecio alkaloids; primary liver tumours in rats as a result of treatment with (1) a mixture of alkaloids from S. jacobaea Lin.; (2) retrorsine; (3) isatidine.. Br J Cancer.

[OCR_00645] WILSON J. W. (1954). Hepatomas produced in mice by feeding betonite in the diet.. Ann N Y Acad Sci.

